# Supplementary material for: Regulation of DNA (de)Methylation Positively Impacts Seed Germination during Seed Development under Heat Stress
Source: Genes (Basel). 2021 Mar 23;12(3):457. doi: 10.3390/genes12030457 (PMC8005211; doi:10.3390/genes12030457)
Supplement: Supplementary file 1 [file genes-12-00457-s001.zip › Supplementary-Figure-1.pdf]

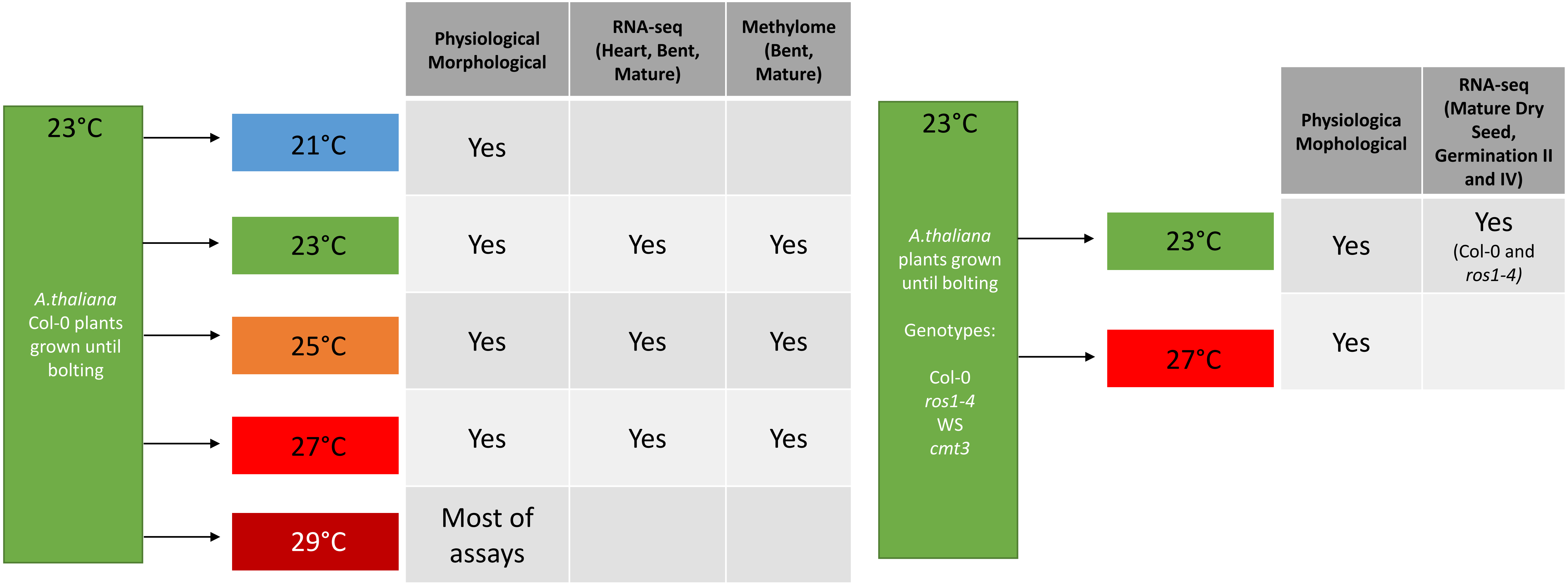


RNA-seq and Methylome
Gene Expression Omnibus
(GEO, GSE167245)
Malabarba et al., 2021 (this study)

Data Mining RNA-seq
Gene Expression Omnibus
(GEO, GSE94459)
Narsai et al., 2017
