# Supplementary figures and images for: Regulation of DNA (de)Methylation Positively Impacts Seed Germination during Seed Development under Heat Stress
Source: Genes (Basel). 2021 Mar 23;12(3):457. doi: 10.3390/genes12030457 (PMC8005211; doi:10.3390/genes12030457)

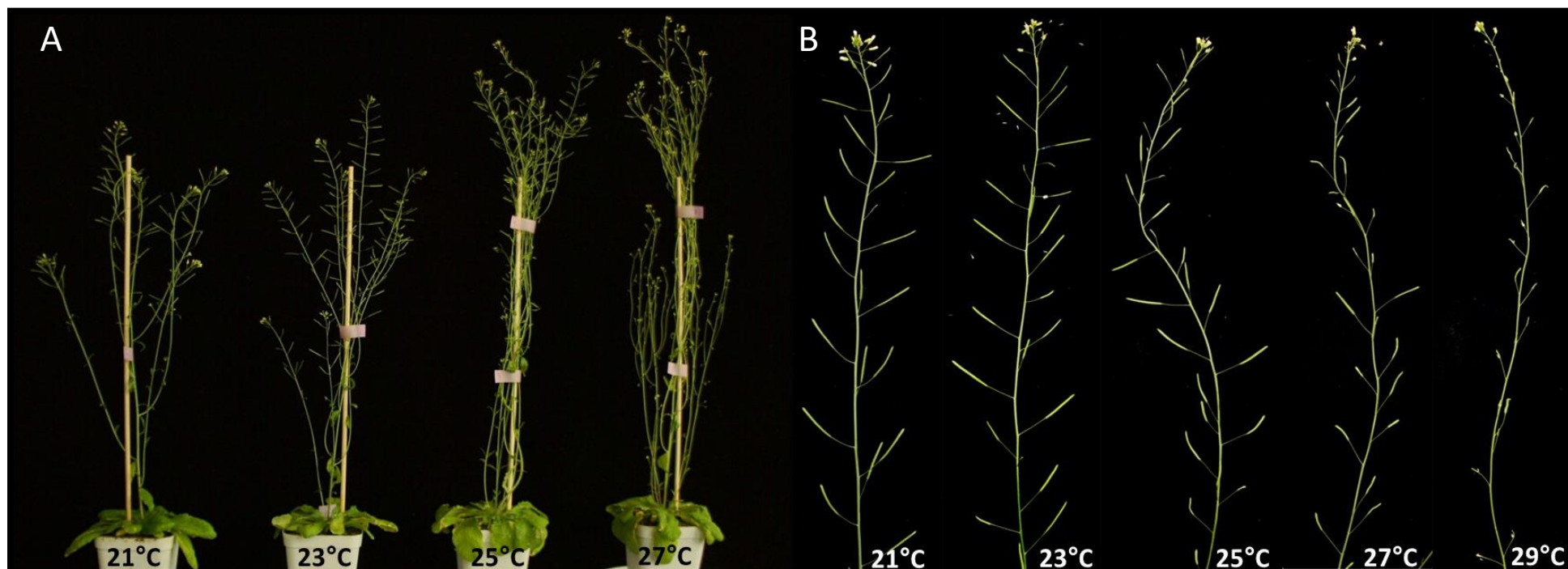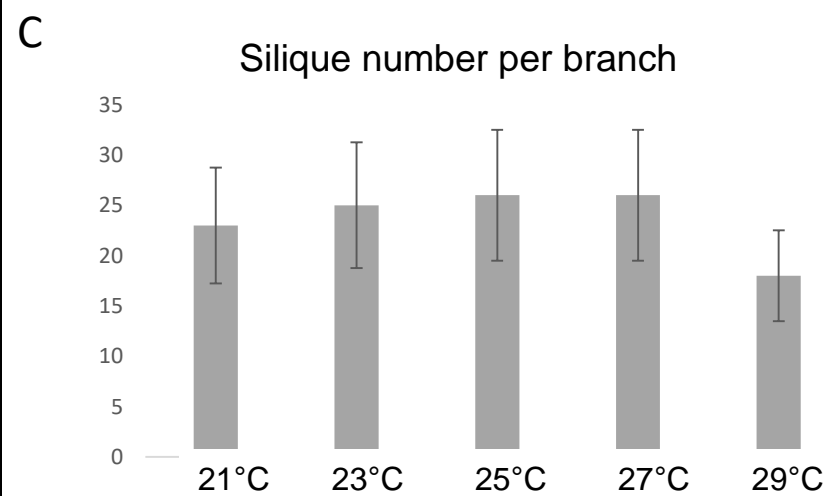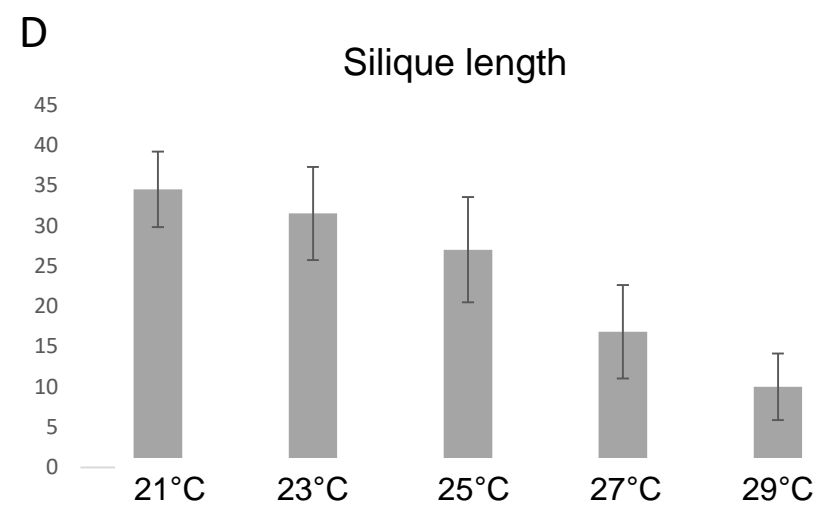

Supplement: Supplementary file 1 [file genes-12-00457-s001.zip › Supplementary-Figure-2.pdf]

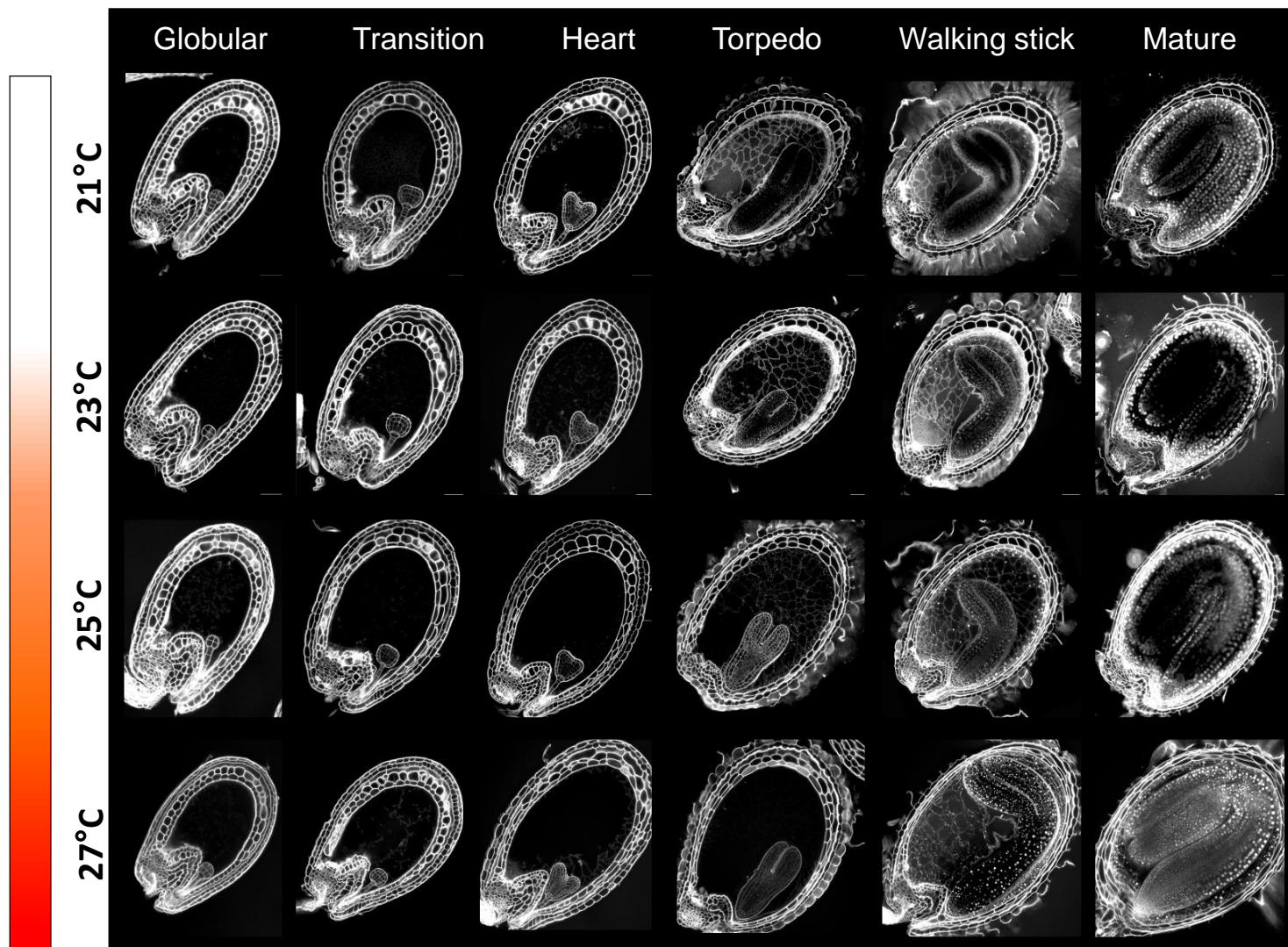

Supplement: Supplementary file 1 [file genes-12-00457-s001.zip › Supplementary-Figure-3.pdf]

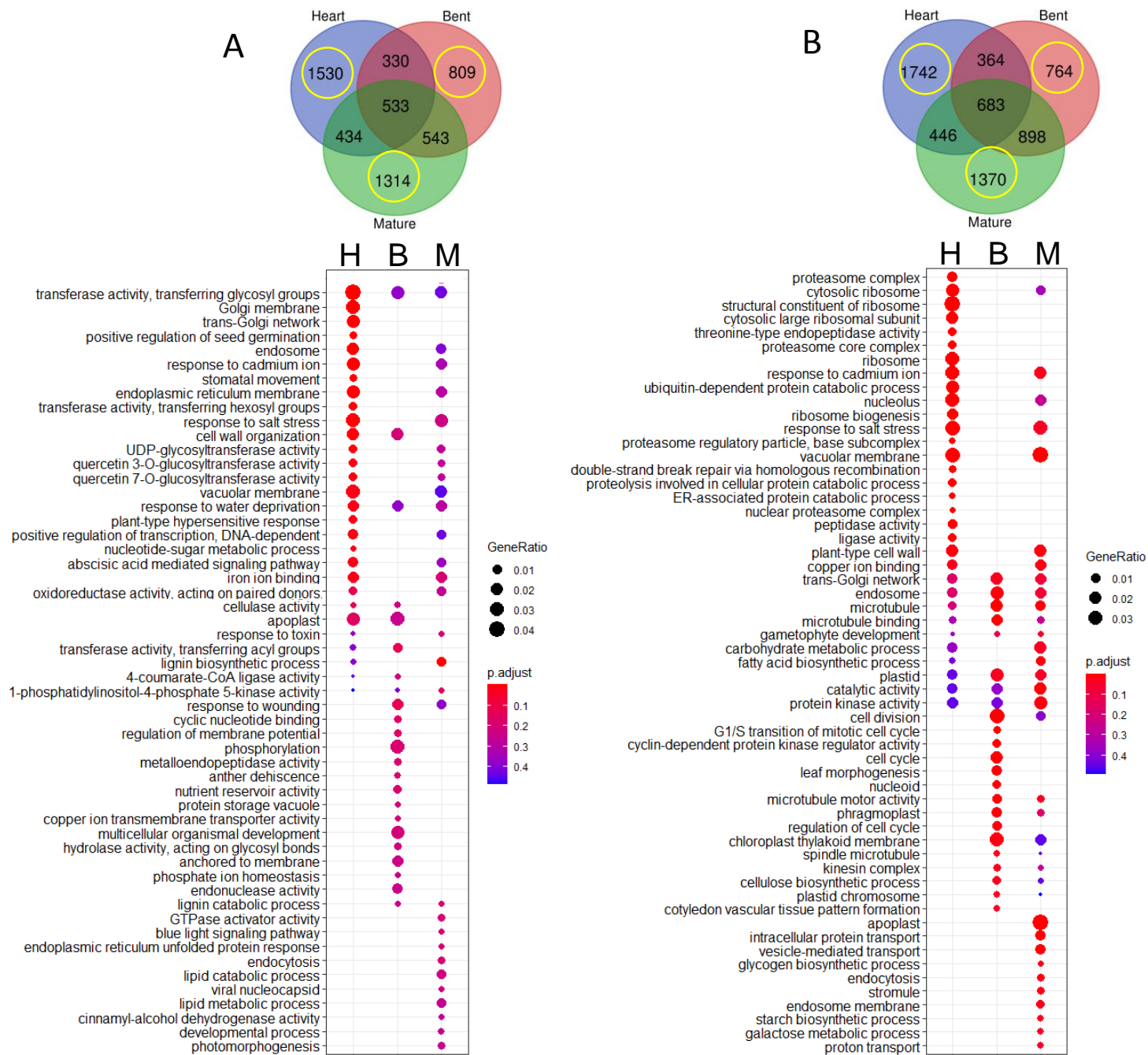

Supplement: Supplementary file 1 [file genes-12-00457-s001.zip › Supplementary-Figure-4.pdf]
